# Evaluating serum S-Equol, indoxyl sulfate, and TMAO in predicting urinary stones in children: a prospective study

**DOI:** 10.1007/s00240-025-01737-w

**Published:** 2025-04-05

**Authors:** Aylin Gencler, Hakim Celik, Abit Demir

**Affiliations:** 1 Department of Pediatric Nephrology, Harran University Faculty of Medicine, Şanlıurfa, Turkey; 2 Department of Medical Physiology, Harran University Faculty of Medicine, Şanlıurfa, Turkey; 3 Department of Pediatrics, Harran University Faculty of Medicine, Şanlıurfa, Turkey

**Keywords:** Urinary stone disease, Pediatric nephrolithiasis, S-equol, Indoxyl sulfate, Trimethylamine N-oxide, Gut microbiota, Metabolites

## Abstract

Gut microbiota is vital in maintaining health and has been implicated in urinary stone disease. Patients with and without stones have different microbial compositions. In this context, we assessed serum levels of S-equol, indoxyl sulfate (IS), and trimethylamine N-oxide (TMAO), which are metabolites thought to be associated with gut microbiota, and their prognostic values in predicting stone formation in children with urinary stone disease. The study population consisted of children aged between one month and 18 years with urinary stone disease. The patient group consisted of 44 children with urinary stone disease, and the control group consisted of 44 healthy children who were matched with the patient group in terms of age and gender. The study’s primary outcomes were the differences between the groups in serum metabolite levels. Serum S-equol and TMAO levels were significantly lower in the patient group than in the control group. There was no significant difference between the groups in serum IS levels. There were also no significant correlations between serum metabolite levels and age in either group. Children with urinary stone disease had significantly lower serum S-equol and TMAO levels than healthy control subjects, suggesting a possible link between these metabolites and stone formation.

## Introduction

The human gut microbiota plays a crucial role in maintaining health by modulating the immune system, protecting against pathogens, and regulating carbohydrate and lipid metabolism [1]. Alterations in the gut microbiome have been linked to the development of various diseases, including urinary stone disease [2]. Notably, individuals with kidney stones tend to have a predominance of Bacteroides species, whereas Prevotella species are more dominant in healthy controls [3]. Similarly, distinct variations in urinary microbiota composition have been observed between patients with and without urinary stones [4]. Although the exact mechanisms underlying the interaction between gut and urinary microbiota remain unclear, the gut-kidney axis, particularly microbiota profiles, is believed to play a role in the pathophysiology of kidney stone formation [1, 4–6].

Metabolites, the byproducts of metabolic processes, are central to host-microorganism and microorganism-microorganism interactions, influencing overall health and contributing to pathological conditions [6]. Specific metabolites absorbing key compounds such as sodium, calcium, and oxalates may contribute to kidney stone formation [6, 7]. Additionally, bacterial-derived metabolites or biomolecules may promote or inhibit stone formation, although their precise mechanisms remain largely unknown [4].

One such metabolite of interest is S-(−)7-hydroxy-3-(4′-hydroxyphenyl)-chroman, or S-(−) equol (S-equol), a potent phytoestrogen derived from soy isoflavones and produced in the distal intestine and colon. Due to its structural similarity to 17β-estradiol, S-equol exhibits significant biological activity, including selective binding to estrogen receptor β and androgen-modulating effects in vivo, suggesting potential therapeutic applications in hormone-dependent conditions [8, 9]. Studies have highlighted soy’s various metabolic and cardiovascular benefits, particularly those related to S-equol [10, 11]. Moreover, dietary polyphenols have been shown to prevent oxidative damage and reduce urinary crystal concentrations in nephrolithiasis [12–14]. While microbial production of S-equol has been suggested to reduce urinary calcium excretion, its direct association with kidney stone disease remains unclear [6].

Another metabolite, indoxyl sulfate (IS), a gut-derived uremic toxin, has been implicated in glomerular sclerosis and interstitial fibrosis through oxidative stress and inflammatory pathways [10–15]. However, its role in urinary stone formation has yet to be determined.

Trimethylamine N-oxide (TMAO), a metabolite produced via the microbial metabolism of phosphatidylcholine, choline, and L-carnitine, is commonly associated with a Western diet rich in fat and red meat [16]. Elevated TMAO levels have been linked to an increased risk of thrombotic and cardiovascular diseases, as well as kidney dysfunction, including collagen deposition and tubulointerstitial fibrosis [16, 17]. However, its classification as a uremic toxin and its potential role in promoting urinary stone formation remain subjects of ongoing investigation [2].

In light of this background, this study aims to evaluate serum levels of S-equol, IS, and TMAO and assess their prognostic value in predicting stone formation in children with urinary stone disease.

## Materials and methods

### Study design

This prospective case-control study was approved by the local ethics committee (Approval Date: 01.04.2024, Approval Number: HRÜ/24.03.18) and conducted by the principles of the Declaration of Helsinki. Written informed consent was obtained from the legal guardians of all participating children.

### Study population and sample

The study included consecutive children aged one month to 18 years diagnosed with urinary stone disease and having normal renal function, as indicated by normal serum creatinine levels, at Harran University Faculty of Medicine, Department of Child Health and Diseases—a tertiary referral center in Sanliurfa, Turkey—between April 2024 and August 2024. Urinary stone disease was diagnosed by renal ultrasonography and/or documented spontaneous stone passage [18]. Stones retrieved via spontaneous passage or interventional/surgical procedures were analyzed using X-ray diffraction at the Institute of Mineral Inspection and Research Laboratory, Ankara, Turkey.

Exclusion criteria included nephrocalcinosis, neuropathic bladder, major congenital urinary tract anomalies, symptomatic urinary tract infection, hematuria, chronic kidney disease (CKD), prior major reconstructive bladder surgery requiring catheterization, and significant cardiac, pulmonary, gastrointestinal, or neurological conditions [19].

The control group was randomly selected from children attending the same outpatient clinic who had normal renal function and no significant systemic disease.

The minimum required sample size was calculated as 84 participants (42 per group) based on an estimated medium effect size (d = 0.55), a 5% significance level, and 80% power, given the absence of prior comparable studies and the inability to conduct a pilot study. Ultimately, 44 children with urinary stone disease and 44 age- and sex-matched healthy controls were included.

### Data collection

Demographic (age, gender) and clinical (consanguineous marriage, family history of stone disease, history of urinary tract infection) characteristics were collected prospectively. Laboratory tests included a complete blood count, serum creatinine, and blood urea nitrogen measurements.

Sample Collection and Urine Analysis.

Specimens were obtained from all subjects, including both venous blood and spot urine samples. Following collection, blood specimens underwent centrifugation at 3000 rpm for 10 min, with separated sera preserved at -80 °C pending metabolite analysis for S-equol, IS, and TMAO quantification.

Urine analysis included pH, specific gravity, and the calculation of spot urine calcium, citrate, cystine, uric acid, and oxalate-to-creatinine ratios [20]. No dietary restrictions were imposed at the time of urine collection. None of the participants took medication for kidney stones or had a symptomatic urinary tract infection [18].

### S-Equol, IS, and TMAO analyses

Serum S-equol, IS, and TMAO concentrations were measured using the Sandwich Enzyme-Linked Immunosorbent Assay (ELISA) method. Microelisa strip plates pre-coated with analyte-specific antibodies were used. After adding standards or samples, the analytes bound to the antibodies on the plate. A horseradish peroxidase (HRP)-conjugated secondary antibody was then introduced and incubated.

Following incubation, unbound components were washed away, and a tetramethylbenzidine (TMB) substrate solution was added. In the presence of the analyte-HRP complex, the solution produced a blue color. The reaction was halted with a stop solution, changing the color to yellow. The optical density (OD) was measured spectrophotometrically at 450 nm, with OD intensity directly proportional to the analyte concentration.

Analyte concentrations were determined by comparing sample OD values to a standard curve derived from known concentrations [21–24]. The analyses were performed using ELISA kits specific to S-equol (cat. no. SLD4188Hu), IS (cat. no. SL3200Hu), and TMAO (cat. no. SL3802Hu) from Sunlong Biotech Co., Ltd.

### Statistical analysis

The study’s primary outcomes were the differences between the groups in serum S-equol, IS, and TMAO levels. For the statistical presentation, descriptive data were summarized as means ± standard deviations for normally distributed continuous variables, medians with minimum-maximum ranges for non-normally distributed continuous variables, and frequencies with percentages for categorical variables. The normality assessment of variables employed various methodological approaches tailored to data characteristics and sample size. Specifically, we utilized the Shapiro-Wilk test complemented by visual assessment tools including histograms and quantile-quantile (Q-Q) plots for smaller sample groups (*n* < 50), ensuring robust evaluation of distribution patterns.

For between-group categorical variable comparisons, we employed Pearson’s chi-square test when analyzing 2 × 2 contingency tables with expected frequencies ≥ 5, maximizing statistical reliability with adequate sample sizes. For smaller samples or RxC tables with expected cell counts < 5, we utilized the Fisher-Freeman-Halton test to maintain analytical validity.

For continuous variables, we applied the independent samples t-test for normally distributed data and the Mann-Whitney U test for non-normally distributed metrics when comparing the two groups. Correlations between non-normally distributed variables were examined using Spearman’s rank correlation coefficient.

All statistical analyses were performed using Jamovi 2.3.28 (2023, www.jamovi.org) and JASP 0.19.0 (2024, jasp-stats.org). Statistical significance was established at *p* ≤ 0.05 for all assessments.

## Results

When comparing demographic characteristics, patient and control groups exhibited comparable age and gender distributions (*p* = 0.203 and *p* = 0.999, respectively). Despite the higher prevalence of parental consanguinity observed in the patient cohort compared to controls (31.8% versus 15.9%), this difference did not reach statistical significance (*p* = 0.133), as detailed in Table 1.

**Table 1:** Demographic and clinical characteristics of the groups

	Group Stone (*n* = 44)	Group Control (*n* = 44)	*p*
**Age (year)** ^†^	5.5 [1.0–17.0]	5.0 [1.0–16.0]	0.203
**Sex** ^‡^			
Female	20 (45.5)	20 (45.5)	0.999
Male	24 (54.5)	24 (54.5)	
**Consanguineous marriage** ^‡^	14 (31.8)	7 (15.9)	0.133

^†^: Median [Min-Max], ^‡^: n (%)

Table 2 presents the characteristics of urinary stones, including localization, size, and number, as well as the results of stone composition analyses. The median maximum stone diameter was 5.54 mm, and solitary kidney stones were detected in 37 children (84.1%). Five (62.5%) had calcium oxalate stones among the eight children who underwent stone analysis.

**Table 2:** Clinical characteristics of children with urinary stones

	Group Stone (*n* = 44)
**Maximum stone diameter (mm)** ^†^	5.5 [3.0–15.0]
**Number of stones** ^†^	2.0 [1.0–6.0]
**Children with single stone** ^‡^	11 (25.0)
**Stone localization** ^‡^	
Kidneys	37 (84.1)
Ureters	3 (6.8)
Urinary bladder	2 (4.5)
Kidney + ureter	1 (2.3)
Kidney + urinary bladder	1 (2.3)
**Type of stones (** ***n*** ** = 8)** ^‡^	
Calcium oxalate	5 (62.5)
Hypoxanthine	1 (12.5)
Cystine	2 (25.0)

^†^: Median [Min-Max], ^‡^: n (%)

Nearly half of the children with urinary stones (*n* = 21, 47.7%) had a familial history of urinary stone disease. Additionally, 28 children (63.6%) had recurrent urinary tract infections, and 17 (38.6%) had a history of recurrent urinary stones.

The results of the laboratory tests are summarized in Table 3. A significant difference was observed between the groups in serum chloride levels (*p* < 0.001). However, categorical analysis of chloride levels did not reveal a statistically significant difference (*p* = 0.374). No other significant differences between the groups were found in serum laboratory parameters (*p* > 0.05).

**Table 3:** The results of laboratory investigations of the study groups

	Group Stone (*n* = 44)	Group Control (*n* = 44)	*p*
**Blood**			
Sodium (mmol/L) ^§^	138.9 ± 1.9	138.1 ± 2.5	0.077
Potasium (mmol/L) ^§^	4.3 ± 0.5	4.2 ± 0.3	0.154
Calcium (mg/dL) ^§^	9.8 ± 0.6	9.7 ± 0.4	0.833
Phosphorus (mg/dL) ^†^	4.9 [2.0–7.3]	4.8 [3.5–6.7]	0.792
Magnesium (mg/dL) ^†^	2.0 [1.0–2.5]	2.0 [1.7–2.9]	0.351
Creatinine (mg/dL) ^†^	0.4 [0.2–1.1]	0.3 [0.2–0.8]	0.657
Blood urea nitrogen (mg/dL) ^§^	22.1 ± 7.8	22.6 ± 5.6	0.746
Albumin (mg/dL) ^†^	4.5 [3.7–5.2]	4.5 [4.0–5.1]	0.575
Uric acid (mg/dL) ^†^	4.0 [0.5–9.6]	3.8 [1.9–5.3]	0.332
Vitamin D (ng/mL) ^†^	24.5 [5.4–80.0]	25.5 [8.6–56.0]	0.924
Parathyroid hormone (pg/mL) ^†^	34.4 [12.0–231.4]	36.9 [23.0–72.4]	0.317
Chloride (mmol/L) ^§^	102.4 ± 3.1	105.8 ± 2.8	**< 0.001**
Chloride groups ^‡^			
Low	1 (2.3)	0 (0.0)	0.374
Normal	41 (95.3)	40 (90.9)	
High	1 (2.3)	4 (9.1)	
**Urine**			
pH ^†^	6.0 [5.5–8.0]	6.5 [6.0–7.0]	**0.036**
Specific gravity (g/mL) ^†^	1010.0 [1003.0–1027.0]	1010.0 [1002.0–1019.0]	0.198
Calcium/creatinine (mg/mg) ^†^	0.16 [0.01–2.10]	0.03 [0.01–0.17]	**< 0.001**
Citrate/creatinine (mg/mg) ^†^	1.25 [0.14–7.30]	1.80 [0.98–2.67]	**0.043**
Cystine/creatinine (mg/mg) ^†^	0.02 [0.01–2.50]	0.01 [0.01–0.03]	**0.028**
Uric acid/creatinine (mg/mg) ^†^	0.40 [0.10–1.00]	0.06 [0.04–0.30]	**0.004**
Oxalate/creatinine (mg/mg)	0.04 [0.01–1.20]	0.01 [0.01–0.04]	**< 0.001**

^§^: Mean ± Standard Deviation, ^†^: Median [Min-Max], ^‡^: n (%)

Bold p-values indicate statistical significance (*p* ≤ 0.05)

Urine pH and composite urinary parameters differed significantly between the groups (*p* < 0.05). Children with urinary stones had significantly lower urine pH than healthy controls (*p* = 0.026). Additionally, median urinary ratios of calcium/creatinine, citrate/creatinine, cystine/creatinine, uric acid/creatinine, and oxalate/creatinine were significantly higher in the patient group than in the control group (*p* < 0.05) (Table 3).

**Fig. 1:**
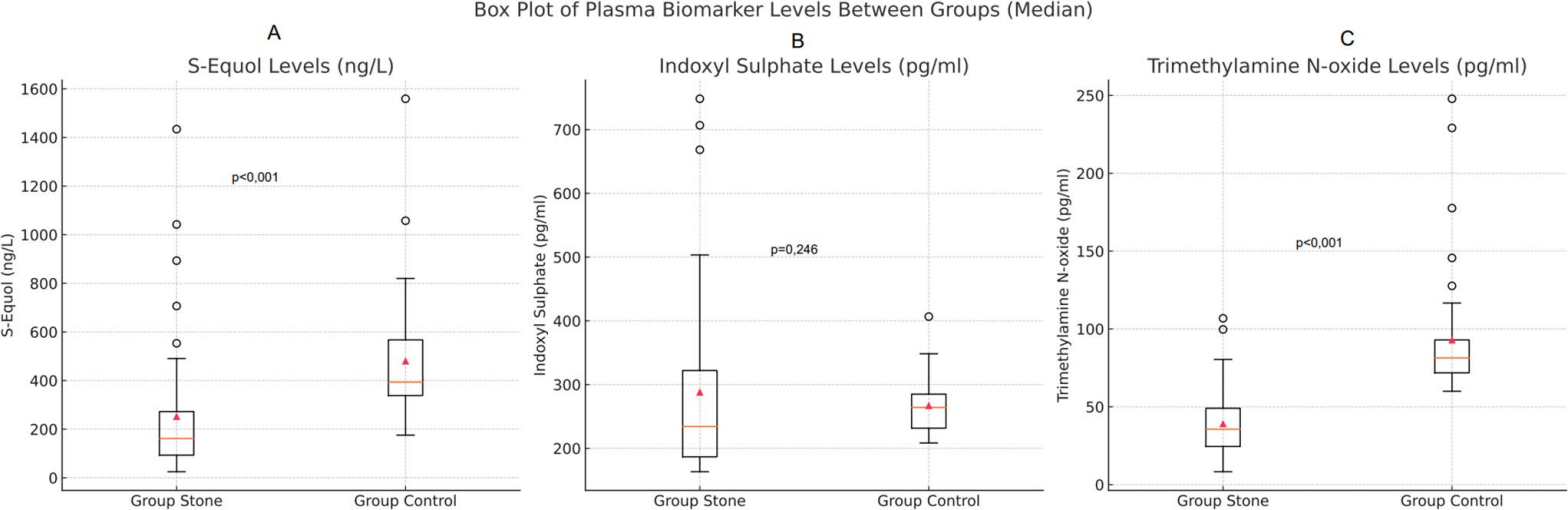
Schematic representation of serum (**A**) S-equol, (**B**) indoxy sulphate, and (**C**) trimethylamine N-oxide levels in the groups

Serum S-equol and TMAO levels were significantly lower in the patient group than in the control group (*p* < 0.001). In contrast, the groups had no significant difference in serum IS levels (*p* = 0.246) (Table 4; Fig. 1).

**Table 4:** Plasma S-equol, Indoxy sulphate, and trimethylamine N-oxide levels of the groups

	Group Stone (*n* = 44)	Group Control (*n* = 44)	*p*
S-equol (ng/L) ^†^	161.9 [25.0–1434.2]	393.5 [175.4–1559.2]	**< 0.001**
Indoxyl sulphate (pg/ml) ^†^	234.2 [163.3–748.3]	264.2 [208.3–406.7]	0.246
Trimethylamine N-oxide (pg/ml) ^†^	35.6 [8.3–106.8]	81.4 [60.0–247.8]	**< 0.001**

^†^: Median [Min-Max]

Bold p-values indicate statistical significance (*p* ≤ 0.05)

Serum levels of S-equol, IS, and TMAO showed no significant correlation with age in either group (*p* > 0.05) (Table 5).

**Table 5:** Correlation of serum S-equol, Indoxy sulphate, and trimethylamine N-oxide levels with age of the children in the groups

		Group Stone (*n* = 44)	Group Control (*n* = 44)
		Age	Age
S-equol	r	-0.189	-0.107
	p	0.219	0.488
Indoxyl sulphate	r	0.046	0.027
	p	0.766	0.864
Trimethylamine N-oxide	r	-0.131	-0.048
	p	0.395	0.759

## Discussion

This study demonstrated that children with urinary stone disease had significantly lower serum S-equol and TMAO levels than healthy children. Considering the potential role of these molecules in urinary stone formation, S-equol and TMAO-related metabolic changes may contribute to stone formation. However, further studies are needed to explore the underlying pathophysiological mechanisms.

Polyphenols, particularly catechins found in green tea, have been reported to inhibit kidney stone formation in experimental models [12, 25]. Although flavonoids, a subgroup of polyphenols, have known health benefits, no clinical data exist regarding the impact of dietary flavonoids—especially soy flavanones and their microbial metabolite, S-equol—on urinary stone development [12, 13]. This study is the first to investigate this potential association.

While urine is a key marker of systemic isoflavone exposure in children, previous studies found no significant correlation between urinary and blood isoflavone concentrations [26]. This suggests that urinary concentrations may not accurately reflect systemic bioavailability, with blood or saliva possibly serving as better indicators [27, 28]. Given these considerations, we chose to measure serum S-equol levels. However, future research should assess urinary and serum levels of multiple metabolites to provide a more comprehensive understanding of isoflavone metabolism.

The “equol producer” status, defined by a log-transformed equol/daidzein ratio of − 1.75 or higher in urine, denotes individuals who can produce S-equol after soy consumption [29]. This ability is influenced by genetic factors, maternal and environmental influences, and diet during early life [8, 29]. Significant geographical differences have been reported, with over 50% of adults in Japan, China, and Korea being equol producers, compared to only 25–35% in Western populations [8, 9].

The relationship between maternal and childhood equol production remains unclear. A study in Japan found that 35.5% of mothers and only 13.8% of their children were equol producers, suggesting maternal influence [29]. However, our study did not assess equol production frequency or maternal dietary factors. While it has been hypothesized that serum S-equol levels should increase with age due to greater soy flavonoid intake, we found no correlation between serum S-equol levels and age in either the patient or control group. Future studies should consider dietary intake, equol production status, and maternal influences to clarify their roles in stone disease development.

There is also some ambiguity about the relationship of equol production between children aged 5–7 years and their mothers. In a study investigating the relationship between equol producer status among Japanese mothers and their children, Wada et al. [29]found that 35.5% of mothers and 13.8% of their children were equol producers, suggesting a potential maternal influence on equol production in children. This study did not assess equol production frequency or maternal factors affecting equol production. Although it has been anticipated that serum levels of S-equol, which promotes equol production due to higher consumption of soy flavonoids, would increase with age, our results showed no correlation between serum S-equol levels and age in either the patient or control group. Therefore, future studies should better consider maternal factors, dietary intake, and equol production status to understand their roles in the development of stone disease.

Contradictory findings exist regarding the relationship between dietary isoflavone intake and equol production [9, 29]. While Wada et al. [29] found no significant association between dietary isoflavone intake and urinary flavonoid metabolites, Sosvorová et al. [9] reported that urinary S-equol levels increased after three months of phytoestrogen therapy. Additionally, a prospective study showed that S-equol was undetectable in the urine of 98% of breastfed infants, 95% of soy formula-fed infants, and 78% of cow’s milk formula-fed infants [26]. In the same study, S-equol concentrations in blood and saliva were below detection limits in all infant groups. Similarly, we observed significantly lower serum S-equol levels in children with urinary stones, warranting further investigation into its role in stone formation.

Prior studies have reported elevated serum TMAO levels in patients with chronic kidney disease (CKD), particularly in diabetic individuals [16, 17]. Higher TMAO levels correlate with age, serum creatinine, and urinary albumin-to-creatinine ratio, with an inverse correlation with the estimated glomerular filtration rate (eGFR) [17]. Dong et al. [30] also demonstrated that TMAO promoted calcium oxalate deposition in hyperoxaluria.

In contrast to these findings, our study revealed lower serum TMAO levels in children with urinary stones. Although we did not measure urinary TMAO levels, they might be elevated, considering TMAO’s known role as a uremic toxin. Prospective studies evaluating blood and urinary TMAO levels are needed to clarify its role in urinary stone formation further.

As a protein-bound, gut-derived uremic toxin, IS has been associated with reduced renal clearance and increased circulating levels in CKD [2, 10]. While we did not measure urinary IS levels, our study found comparable serum IS levels between children with and without urinary stones, providing no strong evidence for its role in stone formation. Future studies should investigate urinary IS concentrations to determine their potential contribution to systemic IS levels in children with and without urinary stone disease.

The relationship between serum metabolites and urinary chemistry in kidney stone disease remains interesting. While our study evaluated serum concentrations of S-equol, indoxyl sulfate (IS), and trimethylamine N-oxide (TMAO), their potential effects on urinary composition warrant further investigation. Given their roles in gut microbiota metabolism and systemic inflammation, these metabolites could influence the renal handling of stone-associated ions. For instance, TMAO has been linked to altered renal function and oxidative stress, potentially creating an environment conducive to stone formation. However, the impact of renal parenchymal and tubulointerstitial pathological changes on urinary biochemical disturbances related to TMAO remains debated [16, 17].

IS, a gut-derived uremic toxin, has been associated with endothelial dysfunction and renal tubular injury, which could hypothetically affect urinary oxalate excretion [10–15]. S-equol appears to be the most speculative among these metabolites regarding its potential effects on urinary biochemistry. Reduced urinary calcium excretion is the only metabolic change definitively linked to equol [6]. Therefore, microbial alterations due to dysbiosis could, in turn, influence the metabolism and excretion of various urinary metabolites, particularly oxalate and calcium. While this hypothesis is compelling, direct evidence connecting serum levels of S-equol, IS, or TMAO to urinary oxalate excretion is currently lacking.

### Limitations of the study

This study’s primary limitation was its cross-sectional design, restricting our ability to infer causality. Secondly, we only focused on the serum levels of S-equol, TMAO, and IS, did not analyze 24-hour urine excretions, and did not correlate the results of these analyses with detailed dietary records, which prevented us from reaching more definitive conclusions. Additionally, since imaging was not performed to detect kidney stones in the control group, whether these children had kidney stones remains unknown. We can only confirm that they did not have clinically evident stone passage. Our relatively small sample size, the fact that we did not assess the equol producer status of children, and the limited generalizability of our findings due to genetic and environmental heterogeneities may be other limitations of our study. Future large-scale studies with more diverse populations are needed to corroborate our findings.

## Conclusion

In conclusion, our findings suggest a potential link between serum S-equol and TMAO levels and urinary stone disease in children, underscoring the importance of further research into the metabolic factors contributing to kidney stone formation. Understanding the relationships between metabolic factors and stone formation may lead to developing novel diagnostic and therapeutic approaches to managing this condition in pediatric populations.

## Data Availability

No datasets were generated or analysed during the current study.

## References

[1] Chen YY, Chen DQ, Chen L et al (2019) Microbiome-metabolome reveals the contribution of gut-kidney axis on kidney disease. J Transl Med 17(1). 10.1186/s12967-018-1756-4 10.1186/s12967-018-1756-4 PMC6317198 30602367

[2] Mahmoodpoor F, Rahbar Saadat Y, Barzegari A, Ardalan M, Zununi Vahed V S (2017) The impact of gut microbiota on kidney function and pathogenesis. Biomed Pharmacother 93:412–419. 10.1016/j.biopha.2017.06.066 28654798 10.1016/j.biopha.2017.06.066

[3] Stern JM, Moazami S, Qiu Y et al (2016) Evidence for a distinct gut Microbiome in kidney stone formers compared to non-stone formers. Urolithiasis 44(5):399–407. 10.1007/s00240-016-0882-9 27115405 10.1007/s00240-016-0882-9 PMC8887828

[4] Zampini A, Nguyen AH, Rose E, Monga M, Miller AW (2019) Defining Dysbiosis in Patients with Urolithiasis. Scientific Reports 9(1):1–13. 10.1038/s41598-019-41977-6 10.1038/s41598-019-41977-6 PMC6443657 30932002

[5] Caggiano G, Cosola C, Di Leo V, Gesualdo M, Gesualdo L (2020) Microbiome modulation to correct uremic toxins and to preserve kidney functions. Curr Opin Nephrol Hypertens 29(1):49–56. 10.1097/MNH.0000000000000565 31725010 10.1097/MNH.0000000000000565

[6] Miller AW, Penniston KL, Fitzpatrick K, Agudelo J, Tasian G, Lange D (2022) Mechanisms of the intestinal and urinary microbiome in kidney stone disease. Nature Reviews Urology 19(12):695–707. 10.1038/s41585-022-00647-5 10.1038/s41585-022-00647-5 PMC11234243 36127409

[7] Mitchell T, Kumar P, Reddy T et al (2018) Dietary oxalate and kidney stone formation. Am J Physiol Ren Physiol 316(3):F409. 10.1152/ajprenal.00373.2018 10.1152/ajprenal.00373.2018 PMC6459305 30566003

[8] Brown NM, Galandi SL, Summer SS et al (2014) S-(–) equol production is developmentally regulated and related to early diet composition. Nutr Res 34(5):401. 10.1016/j.nutres.2014.03.005 24916553 10.1016/j.nutres.2014.03.005 PMC5027651

[9] Sosvorová L, Mikšátková P, Bičíková M, Kaňová N, Lapčík O (2012) The presence of monoiodinated derivates of Daidzein and genistein in human urine and its effect on thyroid gland function. Food Chem Toxicol 50(8):2774–2779. 10.1016/j.fct.2012.05.037 22659465 10.1016/j.fct.2012.05.037

[10] Matsumoto T, Kojima M, Takayanagi K, Taguchi K, Kobayashi T (2020) Role of S-Equol, indoxyl sulfate, and trimethylamine N-Oxide on vascular function. Am J Hypertens 33(9):793–803. 10.1093/ajh/hpaa053 32300778 10.1093/ajh/hpaa053 PMC7481967

[11] Hazim S, Curtis PJ, Schär MY et al (2016) Acute benefits of the microbial-derived isoflavone metabolite equol on arterial stiffness in men prospectively recruited according to equol producer phenotype: a double-blind randomized controlled trial. Am J Clin Nutr 103(3):694–702. 10.3945/ajcn.115.125690 26843154 10.3945/ajcn.115.125690 PMC4763500

[12] Hefer M, Huskic IM, Petrovic A et al (2023) A mechanistic insight into beneficial effects of polyphenols in the prevention and treatment of nephrolithiasis: evidence from recent in vitro studies. Cryst 2023 13(7):1070. 10.3389/fphar.2022.806470

[13] Rudrapal M, Khairnar SJ, Khan J et al (2022) Dietary polyphenols and their role in oxidative Stress-Induced human diseases: insights into protective effects, antioxidant potentials and Mechanism(s) of action. Front Pharmacol 13. 10.3389/fphar.2022.806470 10.3389/fphar.2022.806470 PMC8882865 35237163

[14] Peerapen P, Thongboonkerd V (2021) Kidney stone proteomics: an update and perspectives. Expert Rev Proteom 18(7):557–569. 10.1080/14789450.2021.1962301 10.1080/14789450.2021.1962301 34320328

[15] Hobson S, Arefin S, Rahman A et al (2023) Indoxyl sulphate retention is associated with microvascular endothelial dysfunction after kidney transplantation. Int J Mol Sci 24(4). 10.3390/ijms24043640 10.3390/ijms24043640 PMC9960432 36835051

[16] Andrikopoulos P, Aron-Wisnewsky J, Chakaroun R et al (2023) Evidence of a causal and modifiable relationship between kidney function and circulating trimethylamine N-oxide. Nature Communications 14(1):1–18. 10.1038/s41467-023-39824-4 10.1038/s41467-023-39824-4 PMC10511707 37730687

[17] Kalagi NA, Thota RN, Stojanovski E, Alburikan KA, Garg ML (2023) Plasma trimethylamine N-Oxide levels are associated with poor kidney function in people with type 2 diabetes. Nutrients 15(4):812. 10.3390/nu15040812 36839170 10.3390/nu15040812 PMC9960644

[18] Demirtas F, Çakar N, Özçakar ZB, Akıncı A, Burgu B, Yalçınkaya F (2024) Risk factors for recurrence in pediatric urinary stone disease. Pediatr Nephrol 39(7):2105–2113. 10.1007/s00467-024-06300-0 38273078 10.1007/s00467-024-06300-0 PMC11147915

[19] Kovacevic L, Lu H, Kovacevic N, Thomas R, Lakshmanan Y, Cystatin C (2020) Neutrophil Gelatinase-associated Lipocalin, and lysozyme C: urinary biomarkers for detection of early kidney dysfunction in children with urolithiasis. Urology 143, 221–226. doi: 10.1016/j.urology.2020.05.050 32505622 10.1016/j.urology.2020.05.050

[20] Paccaud Y, Rios-Leyvraz M, Bochud M et al (2020) Spot urine samples to estimate 24-hour urinary calcium excretion in school-age children. Eur J Pediatr 179(11):1673–1681. 10.1007/s00431-020-03662-z 32388721 10.1007/s00431-020-03662-z

[21] Coulbault L, Laniepce A, Segobin S, Boudehent C, Cabé N, Pitel AL (2022) Trimethylamine N-Oxide (TMAO) and indoxyl sulfate concentrations in patients with alcohol use disorder. Nutrients 14(19). 10.3390/nu14193964 10.3390/nu14193964 PMC9572718 36235617

[22] Duan S, Pi J, Wang CH et al (2022) Assessment of ELISA-based method for the routine examination of serum indoxyl sulfate in patients with chronic kidney disease. Heliyon 8(12). 10.1016/j.heliyon.2022.e12220 10.1016/j.heliyon.2022.e12220 PMC9801083 36590542

[23] Aksoyalp ZS, Erdogan BR, Aksun S (2023) Optimization of enzyme-linked immunosorbent assay kit protocol to detect trimethylamine N-oxide levels in humans. EXCLI J 22:263. 10.17179/excli2022-5617 37575362 10.17179/excli2022-5617 PMC10415589

[24] Setchell KDR, Cole SJ (2006) Method of defining equol-producer status and its frequency among vegetarians. J Nutr 136(8):2188–2193. 10.1093/jn/136.8.2188 16857839 10.1093/jn/136.8.2188

[25] Ye T, Yang X, Liu H et al (2021) Theaflavin protects against oxalate calcium-induced kidney oxidative stress injury via upregulation of SIRT1. Int J Biol Sci 17(4):1050. 10.7150/ijbs.57160 33867828 10.7150/ijbs.57160 PMC8040307

[26] Cao Y, Calafat AM, Doerge DR et al (2009) Isoflavones in urine, saliva, and blood of infants: data from a pilot study on the estrogenic activity of soy formula. J Expo Sci Environ Epidemiol 19(2):223–234. 10.1038/jes.2008.44 18665197 10.1038/jes.2008.44 PMC2630504

[27] Setchell KDR, Zimmer-Nechemias L, Cai J, Heubi JE (1998) Isoflavone content of infant formulas and the metabolic fate of these phytoestrogens in early life. Am J Clin Nutr 68(6 Suppl). 10.1093/ajcn/68.6.1453S 10.1093/ajcn/68.6.1453S 9848516

[28] Franke AA, Custer LJ, Hundahl SA (2004) Determinants for urinary and plasma isoflavones in humans after soy intake. Nutr Cancer 50(2):141–154. 10.1207/s15327914nc5002_3 15623460 10.1207/s15327914nc5002_3

[29] Wada K, Ueno T, Uchiyama S et al (2017) Relationship of equol production between children aged 5–7 years and their mothers. Eur J Nutr 56(5):1911–1917. 10.1007/s00394-016-1233-x 27256298 10.1007/s00394-016-1233-x

[30] Dong F, Jiang S, Tang C et al (2022) Trimethylamine N-oxide promotes hyperoxaluria-induced calcium oxalate deposition and kidney injury by activating autophagy. Free Radic Biol Med 179:288–300. 10.1016/j.freeradbiomed.2021.11.010 34767921 10.1016/j.freeradbiomed.2021.11.010

